# Bluetongue virus spread in Europe is a consequence of climatic, landscape and vertebrate host factors as revealed by phylogeographic inference

**DOI:** 10.1098/rspb.2017.0919

**Published:** 2017-10-11

**Authors:** Maude Jacquot, Kyriaki Nomikou, Massimo Palmarini, Peter Mertens, Roman Biek

**Affiliations:** 1 College of Medical, Veterinary and Life Sciences, Institute of Biodiversity, Animal Health and Comparative Medicine, Boyd Orr Centre for Population and Ecosystem Health, University of Glasgow, Glasgow, UK; 2 MRC-University of Glasgow Centre for Virus Research, Glasgow, UK; 3 The Pirbright Institute, Pirbright, Woking, UK; 4 The School of Veterinary Medicine and Science, University of Nottingham, Sutton Bonington, Leicestershire, UK

**Keywords:** bluetongue, phylogeography, viral diffusion, environmental drivers, predictor testing, vector-borne pathogen

## Abstract

Spatio-temporal patterns of the spread of infectious diseases are commonly driven by environmental and ecological factors. This is particularly true for vector-borne diseases because vector populations can be strongly affected by host distribution as well as by climatic and landscape variables. Here, we aim to identify environmental drivers for bluetongue virus (BTV), the causative agent of a major vector-borne disease of ruminants that has emerged multiple times in Europe in recent decades. In order to determine the importance of climatic, landscape and host-related factors affecting BTV diffusion across Europe, we fitted different phylogeographic models to a dataset of 113 time-stamped and geo-referenced BTV genomes, representing multiple strains and serotypes. Diffusion models using continuous space revealed that terrestrial habitat below 300 m altitude, wind direction and higher livestock densities were associated with faster BTV movement. Results of discrete phylogeographic analysis involving generalized linear models broadly supported these findings, but varied considerably with the level of spatial partitioning. Contrary to common perception, we found no evidence for average temperature having a positive effect on BTV diffusion, though both methodological and biological reasons could be responsible for this result. Our study provides important insights into the drivers of BTV transmission at the landscape scale that could inform predictive models of viral spread and have implications for designing control strategies.

## 1. Introduction

Vector-borne pathogens threaten human and animal health in many parts of the world and are responsible for a high proportion of disease emergence events [1,2]. Theses emergences often involve ecological and environmental drivers, because vector populations are able to respond rapidly to such cues, including shifts in host distribution and climatic as well as landscape characteristics [3]. Understanding the specific environmental factors that drive the emergence and spread of vector-borne pathogens is, therefore, critical for the development of improved control and prevention measures and to reduce disease impacts on human and animal health as well as economic losses.

Bluetongue virus (BTV), an arbovirus, with a segmented double-stranded RNA genome, is the causative agent of ‘bluetongue’, a major disease of ruminants. Host-to-host transmission occurs via vector-competent biting midges in the *Culicoides* spp. complex [4]. Bluetongue outbreaks cause severe economic damage due to the direct effects on livestock [5,6], trade restrictions, and the cost of surveillance and control. In recent decades, Europe has repeatedly experienced numerous BTV incursions of different serotypes, topotypes (regional variants of particular serotypes) and strains [7]. Most incursions occurred either through the eastern Mediterranean or from North Africa through the Iberian peninsula [8,9]. In addition, a BTV-8 strain, thought to be of sub-Saharan origin [10], emerged in 2006 in The Netherlands, resulting in the largest European outbreak to date and causing economic damage of greater than $2 billion [11–13]. Following initial elimination, BTV-8 recently re-emerged in France [14]. Moreover, large areas of southern and eastern Europe continue to be affected by the circulation of both established and newly introduced BTV strains [15,16] with strains commonly undergoing reassortment [17].

Considerable uncertainty remains about the key factors responsible for the emergence, spread and persistence of BTV in Europe. So far, environmental and climatic changes as well as meteorological conditions and events have been suggested [18–20]. This includes distributional changes in the Afro-Asiatic vector *Culicoides imicola*, likely combined with wind-mediated introduction of infected midges. BTV diffusion is expected to be facilitated under conditions that are favourable for midge activity and viral replication. For example, the extrinsic incubation period of the virus is reduced at higher ambient temperature [21], whereas midge flight activity is considerably reduced at higher wind speeds [22,23] and likely higher precipitation too [24]. Moreover, variation in elevation may influence the velocity of BTV spread through its effect on vector movement with open water and higher elevation acting as likely barriers (which could still be overcome through passive wind-mediated dispersal). BTV can infect a wide range of ruminant species including sheep, goat and cattle. However, infection does not always cause clinical signs, raising questions about the role of livestock distribution and abundance in BTV transmission. Identifying which of these factors affect BTV spatial diffusion, and determining their relative importance, is a critical prerequisite for designing interventions and limiting spread.

Recently developed phylogeographic models provide a powerful framework for gaining insights into the diffusion processes of pathogens and their drivers from time-stamped, geo-referenced sequences. One approach to quantify the association between environmental variables and viral lineage movements combines phylogeographic reconstructions in continuous space [25,26] with a novel analytical and statistical framework [27]. However, this approach is unable to deal with environmental data that do not come in a raster-based format, such as information on the prevailing wind direction. A second phylogeographic method incorporates a generalized linear model (GLM) directly into a diffusion model in discrete space to explicitly test for several potential predictors of viral spread [28,29]. However, few studies have examined the robustness of this approach to the chosen level of discretization or assessed its consistency with the continuous approach outlined above.

Here, we apply phylogeographic models to an extensive dataset of BTV genome sequences to determine which factors most affected BTV spread within Europe. More specifically, we apply existing approaches using continuous and discrete state reconstruction to explain heterogeneities of diffusion rates as a function of candidate predictors, including data on climate, environment and host species. Moreover, we extend and evaluate these approaches to address some of their current limitations (incorporation of environmental predictors in non-rasterized format, sensitivity to the level of discretization).

## 2. Material and methods

### (a) Genomic data

**Figure 1.**
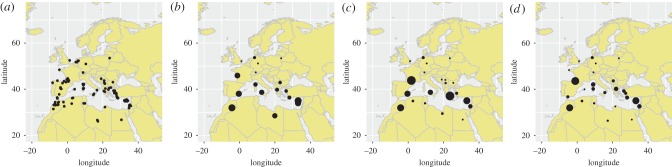
Spatial distribution of samples and discretizations. (*a*) Spatial distribution of the 113 samples used to reconstruct the phylogeographic history of BTV in continuous space and discretizations of these samples in (*b*) arbitrary locations (balanced), (*c*) individual countries or (*d*) geographical zones as described in Materials and methods section. Dots were placed at centroids and their sizes are proportional to the sample size.

We included all the 113 available geo-referenced and time-stamped BTV genomes of isolates that had been collected in European countries, and in countries flanking the Mediterranean Sea, which represent likely source populations of BTV incursions into Europe (figure 1*a*; electronic supplementary material, table S1). Open reading frames for each of the 10 segments within the BTV genome were aligned according to the protein sequence, then converted to codon alignment using ‘PAL2NAL’ [30]. Approximate geo-references (latitude and longitude) were obtained using Google Maps for those cases where only rough meta-data on spatial origin were available (e.g. country, region or city).

### (b) Predictors of bluetongue virus diffusion

We considered several potential predictors of BTV diffusion in Europe that represent relevant climatic, landscape and host-related factors (table 1; electronic supplementary material, figure S1):

**Table 1.** Description of the variables used in this study to explain recent BTV spread in Europe. n.a., not applicable.

predictors	distance measures computed	sources and URLs
GCD	great-circle distance	n.a.
bearing start and endpoints	angles shaped between the direction of the virus diffusion movement and wind directions at both starting and endpoints of this movement	European Centre for Medium-range Weather Forecasts http://apps.ecmwf.int/datasets/data/interim-full-mnth/
precipitation	resistance distances computed when raster treated either as a conductance or resistance factors	European Centre for Medium-range Weather Forecasts http://apps.ecmwf.int/datasets/data/interim
temperature		
wind speed		
mean elevation		Global Multi-resolution Terrain Elevation Data (GMTED 2010) from the United States Geological Survey http://eros.usgs.gov/#Find_Data/Products_and_Data_Available
standard deviation of elevation		
low elevation		derived from mean elevation raster
mid elevation		
high elevation		
terrestrial habitat		derived from cattle density raster
cattle density		Food and Agriculture Organisation http://www.fao.org/ag/AGAInfo/resources/en/glw/GLW_dens.html
sheep density		
goats density		

#### (i) Great circle distances

These were computed with the distVincentyEllipsoid function of the geosphere R package [31].

#### (ii) Climate data

We extracted monthly means of precipitation, temperature, wind speed, and the U and V components of wind (components of the horizontal wind towards the east and north) from the European Centre for Medium-range Weather Forecasts (http://www.ecmwf.int) over 15 years (from January 1998 to December 2012) at a resolution grid of 0.125 × 0.125. Averaged data over the 15 years were generated with the netCDF Operators (NCO) suite of programs (available at http://nco.sourceforge.net). Wind U and V components were used to compute prevailing meteorological wind direction (in degrees) over the 15 years. Based on this, we used the difference in degree angle between the direction of virus diffusion along a branch (see phylogeographic analyses below) and the prevailing wind direction at both starting and end nodes as predictors.

#### (iii) Elevation

Mean and standard deviation of elevation (as a measure for the local variation in elevation) were acquired from the Global Multi-resolution Terrain Elevation Data of the United States Geological Survey (GMTED2010, [32]). GMTED2010 combines the available global elevation data from different public datasets and they are provided as tiles with 30° of longitude × 20° of latitude at a 30-arc-s resolution. The mean elevation raster was used to generate three additional binary rasters. Because the airborne spread of *Culicoides* species and the viral velocity is thought to decrease significantly above 300 m or so [33,34], cells below this elevation were assigned high values of 1000 when corresponding to cells of terrestrial habitat with a mean elevation under 300 m, whereas all other cells were given a value of 0.001 (low elevation). However, members of the *C*. *obsoletus* species complex are commonly found above 1000 m [35]. For two alternative rasters, high values were, therefore, assigned to cells with a mean elevation between 300 and 1000 m (mid elevation), or to the cells above 1000 m (high elevation).

#### (iv) Livestock densities

Modelled densities of three ungulate livestock species (cattle, sheep and goats) were obtained from the Food and Agriculture Organisation at a resolution grid of 0.05 × 0.05 [36].

#### (v) Terrestrial habitat

Information about the distribution of habitat and non-habitat associated with the livestock density data was used to build a binary raster in which cells corresponding to terrestrial areas were assigned a value of 1000 and cells corresponding to open sea a value of 0.001.

Spatial coverage of all environmental rasters was reduced to Europe and the Mediterranean basin with the following boundaries: north = 75.0; south = 20.0; east = −20.0; west = 50.0. All rasters were visualized in QGIS [37] and grid resolutions were reduced to 0.125 × 0.125 to speed up analysis while keeping a sufficient degree of detail. All environmental raster cell values were increased by 1 (except for cells with no data) to avoid cells with values equal to 0. For host density rasters, cells with no data (non-habitat) were assigned the small value of 0.001.

After transformation, resistance distances were computed from rasters using Circuitscape 4.0 [38]. Resistance distances were preferred to least cost path distances because of their ability to accommodate the uncertainty in the route taken by viral lineages. Under this approach, each lineage is considered to have travelled via a random walk between its start and end location as estimated from phylogeographic analyses (see below). Indeed, the computed resistance distance is a graph-theoretic metric based on circuit theory, which takes into account all possible pathways connecting a given pair of locations [38]. Rasters can be treated as either resistance or conductance factors [38], corresponding to the expectation of lower and higher permeability to viral movement associated with this predictor.

### (c) Phylogeography and predictor testing

We reconstructed the spread of BTV in Europe and investigated the potential predictors of virus diffusion by combining information gathered from two phylogeographic analyses. First, BTV phylogeographic history was reconstructed using a continuous space diffusion model and variables were tested *a posteriori* with two statistical approaches*.* Second, we used a discrete phylogeography approach that simultaneously estimates phylogeographic diffusion parameters and the effect of different variables on diffusion, within a GLM framework. Both phylogeographic analyses were done using the BEAST v. 1.8.2 software package (available at http://code.google.com/p/beast-mcmc/), which uses a Bayesian Markov chain Monte Carlo (MCMC) method [39,40]. Further details regarding these analyses are provided below. All BEAST runs were performed using the BEAGLE library to enhance computation speed [41,42]. Convergence of the MCMC outputs was confirmed using Tracer v. 1.6 [43]. Where required, multiple run outputs were combined using LogCombiner v. 1.8.2. and maximum clade credibility (MCC) trees were generated using TreeAnnotator v. 1.8.2 (both provided as part of the BEAST package). Annotated trees were visualized using FigTree v. 1.4.2 (available at http://tree.bio.ed.ac.uk/).

### (d) Phylogeography in continuous space

BTV diffusion dynamics in continuous space were estimated as described in Lemey *et al*. [26]. Unlinked time-scaled phylogenies of BTV segments, with linked evolutionary and demographic models, were simultaneously estimated by combining three chains of 10^9^ steps, sub-sampled every 50 000 generations after discarding 10 per cent of the generations from each, as burn-in. Analyses were performed using a SRD06 model of nucleotide substitutions and an uncorrelated lognormal-relaxed molecular clock under a flexible Bayesian skygrid model as coalescent prior, as described previously [17]. We used the jitter function to add random noise from a fixed window (0.1) to location data for samples with identical geo-references, as recommended by the developers. We compared the homogeneous Brownian diffusion model with random walk (RRW) models, which involve branch-specific scaling factors that are drawn from an underlying distribution (Cauchy, gamma, lognormal). Models were compared using Bayes factors (BFs) based on log marginal likelihoods obtained by path sampling and stepping stone sampling [44–46]. Preliminary investigations revealed that model convergence could not be achieved for more flexible diffusion models (gamma and lognormal, [26]) and that the RWW model under the Cauchy distribution provided a much better fit to the data with very strong support (BF > 500, data not shown). Consequently, only results from the RRW Cauchy model are presented in the following (a summary of the parameter estimates can be found in electronic supplementary material, table S2).

For further analysis, the history of lineage dispersal was recovered from spatially and temporally calibrated phylogenetic trees, in which each internal node has an estimated time and location, allowing for travel times to be calculated for each branch.

#### (i) A posteriori testing of single predictors using SERAPHIM

We investigated the potential of each raster variable to be a predictor of BTV diffusion, when it was treated either as a conductance or resistance factor, using the SERAPHIM library in R [47]. This method is based on the comparison of coefficients of determination, obtained either when travel times are regressed against resistance distances computed from the environmental raster 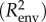, or when travel times are regressed against resistance distances obtained from a null raster 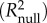. Based on these two values, the *Q* statistic, with 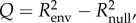 can be calculated. Here, we resampled 100 trees from the BEAST RWW model outputs corresponding to 10 trees per BTV segment. For each predictor, we computed their associated *Q*-values. To assess statistical confidence, we estimated BFs and considered values greater than 3 as evidence of support [48].

#### (ii) A posteriori testing of single predictors and great circle distances using linear regression

**Figure 2.**
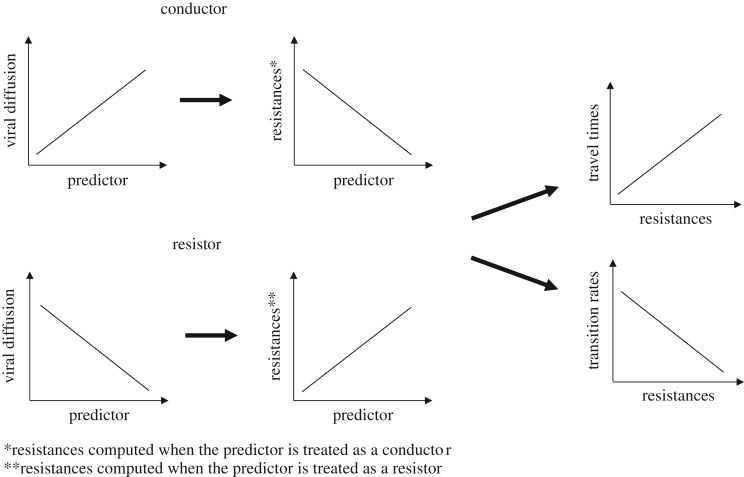
Conceptual diagrams. Graphs show expected relationships for correct specification of a predictor. If a predictor facilitates BTV diffusion, a negative relationship is expected between the predictor when treated as a conductor and computed resistances. If a predictor is acting as a barrier of BTV diffusion, a positive relationship is expected between the predictor when treated as a resistor and computed resistances. For both conductors and resistors, the computed resistances are expected to be positively correlated with the viral travel time (phylogeography in continuous space) and negatively with the transition rates between locations (phylogeography in discrete space).

The SERAPHIM method deals only with variables that can be expressed in a spatial raster in order to compute resistances (or any other distance metric based on a path model). Here, we propose an alternative approach that can accommodate both rasterized and non-rasterized variables. An example of the latter includes the degree angle difference between the direction of the virus diffusion movement and wind directions at both starting and endpoints of the lineages' movement. First, locations and travel times estimated for every branch of the MCC trees of the 10 BTV segments were extracted using the OutbreakTools library in R [49]. For each rasterized predictor, resistances were computed for the corresponding inferred lineage movements. Resistance distances include a spatial component, resulting in resistances that are larger for the points separated by higher geographical distances. In order to make both rasterized and non-rasterized variables comparable, we had to remove the spatial component from the former. To achieve that, we took the residuals from a regression of resistances computed with the predictor against those from a null raster representing spatial distance only. These residuals, thus, represent the variation in environmental distance beyond what we would expect based on spatial distance alone. Locations associated with the parent and descendent nodes of each branch were also used to compute the degree angle difference between the direction of the virus diffusion movement and wind direction at both starting and endpoints of movements. All variables used were log-transformed and standardized to limit the impact of extreme values and to make variables as comparable as possible. Then, travel times were regressed as a function of great circle distances (GCD) and resistance residuals or non-resistance-based predictors: lm(times ∼ GCD + Predictor). As in SERAPHIM, we then used the statistic *Q* to compare the coefficient of determination of each model with the coefficient of the univariate regression of times against GCD only. Different from SERAPHIM, we assessed the statistical support for a predictor based on its significance in the linear model but also its regression coefficient: a variable can only be considered as explanatory if both its associated *Q* value and regression coefficient are positive (figure 2). By contrast, a positive *Q* value combined with a negative coefficient would indicate model misspecification, in that it implies a proposed conductor having a resistance effect or vice versa.

For long branches, representing deep phylogenetic divergences within the tree, spatial inference is potentially unreliable. To examine whether this could have affected our results, we repeated our analysis after excluding all phylogenetic branches with estimated branch lengths greater than 500 years.

A limitation of these approaches is that they allow only one predictor to be assessed at a time. While simultaneous testing of multiple predictors would be desirable and more biologically realistic, the high degree of collinearity between variables (see electronic supplementary material, figure S2) made this problematic. Preliminary analyses involving multiple predictors further revealed that the resulting models were unstable in this case (data not shown). We have, therefore, limited the results presented to those obtained from the univariate approach.

### (e) Phylogeography in discrete space and simultaneous predictors testing

As a complementary approach to continuous phylogeographic inference, we also determined the spatial and temporal dynamics of BTV transmission in Europe in a discrete space [25]. Classification into discrete states contains a level of arbitrariness and sample sizes can strongly impact ancestral reconstruction [29]. We, therefore, analysed patterns of spatial diffusion at three levels of geographical resolution, in order to assess the robustness of the main conclusions with respect to the spatial discretization. In an attempt to include all sequence data while keeping the number of samples per location as balanced as possible, we considered a first partitioning where sequences were arbitrary grouped into 17 locations (figure 1*b*). The second partitioning simply used the country of origin (21 discrete states, figure 1*c*). For the third spatial discretization, we grouped samples in geographical zones. Samples belonging to the same country or to bordering countries were grouped together if they were separated by less than 300 km and not separated by the sea, resulting in 25 different discrete states (figure 1*d*). Spatial discretizations are summarized in electronic supplementary material, table S1. Owing to the very high computation times needed to achieve sufficient mixing and convergence for the analysis in BEAST, we used a set of 500 empirical trees per BTV segment from a previous analysis that did not consider any traits [29].

We used a recent extension of the discrete diffusion approach that simultaneously tests and quantifies potential predictors of the diffusion process in a GLM framework [29,50]. Estimated rates of virus movement among the fixed number of discrete locations are parameterized as a linear function of one or multiple predictors. For each of the three partitioning schemes, we used the cluster centroids to compute distance and resistance matrices to be incorporated in the model. For efficiency and high correlation between variables, we chose to include one representative predictor in the GLM for each kind of variable used in this study: temperature for the meteorological features and the ‘low elevation’ and cattle density for the landscape and livestock components. Resistances were computed when rasters were treated as conductors and again adjusted, keeping residuals of their regression against resistances computed from a null raster. In the end, the model used only included variables reasonably correlated to each other (electronic supplementary material, figure S3). In this case, a resistance predictor correctly specified in the model is expected to be associated with a negative coefficient (figure 2). To control for variation in sample size, it was added as an additional predictor. Following Lemey *et al*. [51], we determined the support for predictors within the model using BFs that are obtained dividing the posterior odds of predictor inclusion by their prior odds. In addition to providing a measure of support for each predictor, the GLM approach also allows the contribution or effect size of each predictor to be quantified by estimating the associated GLM coefficients.

## 3. Results

### (a) Phylogeographic inference in continuous space

#### (i) A posteriori testing of single predictors using SERAPHIM

For each rasterized variable, we estimated *Q*-values, measuring the improvement in the regression fit as a result of including that predictor, for each of the 100 re-sampled trees (10 trees/BTV segment). This generated a distribution of 100 *Q*-values (electronic supplementary material, figure S4) and associated BFs (table 2). None of the predictors showed strong positive or negative associations with BTV movement rates (i.e. BF > 10). However, substantial evidence for associations was seen for livestock density (all species) when treated as conductance factors (i.e. 3 ≤ BF < 10). The standard deviation of elevation, the ‘low elevation’ (elevation below 300 m) and the terrestrial habitat as conductors also showed substantial associations with faster BTV movements (BF ≥ 3.35). When temperature was treated as a conductance factor, 60% of the *Q*-values were positive, but support was low (BF: 1.63). Similarly, 73% of trees had positive *Q*-values when treating precipitation as a resistance factor, but the support was weak (BF: 1.04). For all remaining predictors, no association with lineage movement was evident (BF < 3, less than 50% positive *Q*-values).

**Table 2.** Results of phylogeography in continuous space and *a posteriori* predictor testing.

		SERAPHIM analysis^a^		alternative approach^b^		
	rasters treated as	*Q*^c^ > 0 (%)	BF	*Q*^c^	estimate^d^	*p*-value
bearing start point	n.a.	n.a.	n.a.	+	0.094	6.400 × 10^−3^
bearing endpoint	n.a.	n.a.	n.a.	+	0.093	1.110 × 10^−2^
precipitation	conductance factors	27	0.92	−	−0.002	7.510 × 10^−1^
temperature		60	1.63	+	−0.011	2.070 × 10^−1^
wind speed		30	0.54	+	−0.009	2.290 × 10^−1^
mean elevation		38	1.27	−	0.006	6.530 × 10^−1^
standard deviation of elevation		36	3.55	+	0.188	<2 × 10^−16^
low elevation		39	4.00	+	0.039	1.260 × 10^−6^
mid elevation		15	1.63	+	0.026	4.050 × 10^−3^
high elevation		15	0.75	−	0.005	7.250 × 10^−1^
terrestrial habitat		28	3.35	+	0.089	<2 × 10^−16^
cattle density		28	3.00	+	0.131	<2 × 10^−16^
sheep density		29	3.55	+	0.123	<2 × 10^−16^
goats density		29	3.00	+	0.120	<2 × 10^−16^
precipitation	resistance factors	73	1.04	−	−0.001	8.050 × 10^−1^
temperature		39	0.69	+	−0.010	2.300 × 10^−1^
wind speed		47	1.77	−	−0.001	8.530 × 10^−1^
mean elevation		21	1.70	+	−0.019	9.900 × 10^−2^
standard deviation of elevation		6	0.72	+	−0.206	<2 × 10^−16^
low elevation		1	1.33	+	−0.041	1.830 × 10^−14^
mid elevation		1	0.56	+	−0.045	7.730 × 10^−16^
high elevation		4	1.38	+	−0.018	2.130 × 10^−2^
terrestrial habitat		6	0.82	+	−0.101	<2 × 10^−16^
cattle density		1	0.75	+	−0.160	<2 × 10^−16^
sheep density		0	2.23	+	−0.169	<2 × 10^−16^
goats density		3	0.89	+	−0.150	<2 × 10^−16^

^a^Percentages of positive *Q-*values and associated BF based on 100 sub-sampled trees (10 per BTV segments) using the SERAPHIM R package.

^b^Sign of *Q*-values, predictors coefficient estimates and associated *p*-values based on MCC trees diffusion histories.

^c^*Q* are coefficients of determination.

^d^Estimate refers to the regression coefficient of the bivariate regression.

n.a., not applicable.

#### (ii) A posteriori testing of single predictors and great circle distances using linear regression

As an alternative to SERAPHIM, we used estimated travelling times extracted from MCC trees of the 10 segments (see electronic supplementary material), to estimate *Q*-values and coefficients for all predictors, by comparing the coefficients of determination of the regression of travel times against GCD with and without inclusion of that predictor (table 2).

With respect to non-rasterized data, we found that BTV travel time decreased with increasing deviation from the prevailing wind direction at the starting and endpoint of virus movement. Specifically, values of *Q* and coefficients for the degree angle difference between wind direction at start and end locations and the direction of virus movement were positive with *p*-values < 0.05, highlighting the association between the predominant wind direction and the direction of BTV spread. Excluding long branches from the analysis gave equivalent results (electronic supplementary material, table S3).

For all rasters for which a BF value higher than 3 had been obtained in the SERAPHIM analysis (table 2), we obtained positive values of *Q* associated with positive coefficients and significant *p*-values (figure 2 for interpretation). This verified that the two methods lead to congruent results and confirmed correct specification of predictors as causing either conductance or resistance. The only exception was ‘mid-elevation’ habitat (elevation between 300 and 1000 m) as a conductor, which received support in our method but not in SERAPHIM.

### (b) Phylogeography in discrete space and simultaneous multiple predictors testing

We assessed the ability of phylogeographic inference in discrete space (MCC trees available upon request), combined with GLM approaches, to evaluate which variables predict the rates of location exchange. This was done for a subset of representative predictors and for three different spatial discretizations: ‘balanced’, ‘countries’ and ‘zones’ (figure 1).

**Figure 3.**
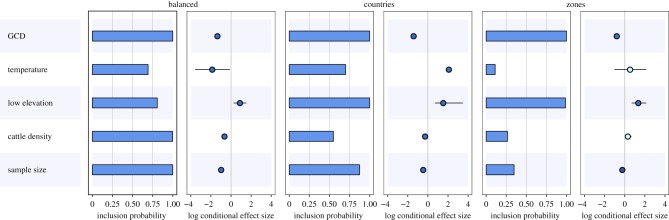
Support and contribution for a subset of predictors of BTV movement between locations for three different spatial discretizations. For each potential predictor, support is represented by an inclusion probability and a relative contribution indicated for log scale GLM coefficients conditional on the predictor being included in the model (posterior mean and 95% Bayesian CI). Darker dots indicate conditional effect sizes supported by Bayes factors greater than 3. For rasterized variables, resistance distances were obtained with raster treated as a conductance factor.

While there was some variability in the results for the three spatial discretizations, some robust patterns emerged (figure 3). There was consistent evidence that GCD between locations negatively influenced the frequency of BTV exchange between locations. The associated log scale conditional effect was high (close to 2) for the three spatial discretizations compared to other inferred coefficients, indicating geographical distance plays a major role in determining rates of BTV movement.

Consistent with results from continuous approaches, viral dispersal occurred significantly more frequently between localities separated by a higher cattle density (variable treated as conductance factor, negative conditional effect size, figure 2 for interpretation) for the ‘balanced’ and ‘countries’ spatial discretizations. No significant effect of the variable was detected at the ‘zones’ level.

In contrast with the continuous trait analysis, results obtained from the three spatial discretizations suggested that BTV spread was limited between locations separated by ‘low-elevation’ habitats (variable treated as a conductance factor, positive conditional effect size). Conversely, while no effect of the temperature was obvious from the continuous approach, a significantly positive effect was seen for two of the discretizations (‘balanced’ and ‘countries’).

For all three spatial discretizations, sample size was identified as an important predictor of BTV exchange between locations.

Summarizing across the different analytical approaches used, our analyses revealed consistent support for BTV diffusion being positively affected by higher livestock densities and low altitude terrestrial habitat (below 300 m) as conductors and by the direction of winds.

## 4. Discussion

This study provides the most comprehensive investigation of BTV incursion and spread in Europe to date. It represents the first time that BTV genetic data have been used to reconstruct the phylogeographic history of the virus and to relate heterogeneity in diffusion process to climate, landscape and host factors. We assessed different methods combining phylogenetic and spatial approaches and found evidence for several predictors affecting BTV diffusion.

### (a) Livestock densities as a key factor in bluetongue virus epidemics

Our results support the idea that densities and distributions of cattle and sheep play a key role in BTV diffusion. Cattle are usually considered a major reservoir of BTV infection. They have been shown to be the preferred target for *Culicoides* biting, possibly because they are larger, emit more semiochemical substances, do not have a woolly coat, and they have detectable viraemia for longer compared to sheep [52]. Initial spread of BTV-8 in The Netherlands in 2006 is thought to have taken place in cattle, before moving more into sheep in 2007 where it caused major losses [53,54]. Consistent with this hypothesis, the re-emergence of the virus in France in 2015 occurred in a cattle production region and only involved a few cases in sheep [14]. Results revealing a considerable role played by sheep are, however, also congruent with a recent work that showed that BTV-1 transmission increased significantly in areas with higher densities of sheep during the epidemic of 2007 in southern Spain [55]. Our results also supported a positive association of BTV spread with the density of goats, which rarely display clinical signs and are generally not considered to play an important role in BTV transmission [56]. However, all resistance distances computed from the three host species densities rasters were highly correlated, which will have limited our ability to distinguish between the effects each individual host species had at this spatial resolution. Applying the approaches used here on data obtained on a smaller spatial scale within a single outbreak would likely be a more fruitful strategy to reveal the role of different types of livestock in BTV spread.

### (b) Marine open water and high altitude as barriers of bluetongue virus diffusion

Our results suggested that BTV spread is facilitated by terrestrial habitat, particularly at low elevation (up to 300 m). These observations are congruent with a barrier effect associated with open ocean and mountains areas, limiting the dispersal of *Culicoides* vectors. While seas and oceans are hostile habitats for the vector, large populations of species in the *Obsoletus* complex can be found in Europe up to 1000 m [35]. However, areas above 300 m failed to be significantly associated with faster viral diffusion in most of our analyses. This suggests that vector populations at these altitudes either have a lower ability to support virus infection and replication (i.e. a lower competence) and/or exhibit a lower ability to transmit BTV in these environments (i.e. a lower capacity) for example due to lower temperatures or higher wind speeds. Further studies on the competence of the *Obsoletus* complex are needed to elucidate the change in vector competence and capacity in relation with landscape features. Conflicting results, suggesting a negative effect of ‘low-elevation’ habitat on BTV movements rates, were obtained in the discrete trait analysis. However, we consider this finding less reliable due to methodological issues seen with this approach, as discussed below.

### (c) Prevailing winds and bluetongue virus long-distance dispersal

We found support for an effect of the prevailing wind directions on BTV diffusion, both at the starting and endpoints of the travel pathway. In the 2006 BTV-8 epidemic in northwest Europe, 2% of infections were inferred to have occurred at distances over 31 km [57,58]. Although other mechanisms such as human transport of infected midges or movement of domestic or wild animals could not be ruled out, our result suggest that these infections are likely to be related to wind dispersal of infected *Culicoides*. Much attention has been given to unusual wind and weather patterns explaining BTV emergence events due to the passive movement of infected vectors (e.g. 2007 in the UK) [59]. The current results suggest that predictive models of BTV spread would benefit from including data on prevailing wind direction, in addition to considering rare weather events.

### (d) Complex role of temperature in bluetongue virus spread

In a 2005 review, Purse *et al*. concluded that host densities and non-climatic abiotic factors are unlikely to be responsible for recent BTV incursions in Europe as these factors had largely remained unchanged during the last century. This, along with mechanistic modelling of BTV transmission risk [20], argues that the repeated emergence of BTV in Europe in recent decades could be related to a general increase in temperature. However, none of our analyses yielded evidence for the role of temperature, considered either as a conductance or resistance factor, in BTV spread in Europe. Multiple non-exclusive lines of argument might explain this result.

First, the relationship between temperature and the parameters affecting BTV transmission is complex and probably nonlinear. BTV transmission is optimal at a mean temperature of 20°C–25°C and decreases at both warmer and cooler temperatures [21,60]. During a 2007 outbreak of BTV-1 in Andalusia for example, there was an overall positive correlation between temperature and basic reproductive number (R0); however, it has been shown that this relationship was not linear [55]. Given these complexities, it might be more appropriate to consider temperature in a nonlinear fashion by examining critical thresholds and classifying resistance values accordingly [61]. This would also require working at a finer temporal scale. In addition, our study encompassed data from 12 different BTV serotypes introduced to Europe over the past 15 years. There could be considerable phenotypic variability among these viruses, with respect to their interactions with different vector species at different temperatures. For instance, during the Spanish 2007 BTV-1 epidemic, involving *C. imicola* as the main vector, the reproductive number fell below one when temperatures dropped below 21°C. By contrast, a much lower threshold of 15°C was reported in the BTV-8 epidemic in Northern Europe in 2007/08 that involved *C. obsoletus* [62]. Applying phylogeographic analyses and test of temperature effects to a single serotype or virus strains might, therefore, be more appropriate and yield a clearer signal.

As an additional factor, climate data in our analysis were summarized over a 15-year window and could have masked potentially important temporal fluctuations. A recent phylodynamic application to relax the time–homogeneity assumption in phylogeographic reconstructions [63] represents a promising possibility for incorporating this temporal heterogeneity in the future.

### (e) Diffusion models and predictor testing methodology

We considered different analytical tools available for phylogeographic analysis of pathogen diffusion, but encountered some methodological challenges and inconsistencies. In the analysis using space as a discrete trait, spatial scale and discretization were shown to have a strong effect on our results, in terms of effect sizes and in some cases even the direction of the effect. Difficulties associated with geographical partitioning in phylodynamic models have previously been noted [29] and our results reinforce this. Using centroid positions to represent clusters, we inherently lose a lot of spatially explicit information that may be informative in explaining the overall diffusion process. This could be particularly problematic at the large geographical scale at which we were working here. Increasing the number of clusters and centroids might help to retain more resolution, but results in smaller sample sizes per cluster and becomes more computationally intensive as it increases model complexity.

Although the use of GLMs for predictor testing of pathogen diffusion has inherent advantages due to the ability to include large numbers of predictors simultaneously, we found that it can suffer from problems of non-independence among the spatial predictors. Multicollinearity among explanatory variables is a well-recognized issue in multivariate regression analyses and our results show that the GLM approach implemented as part of the Bayesian phylogeographic inference in BEAST is no exception to this. An improvement for future work would be to allow the proper co-analysis of several environmental factors in a multivariate framework using, for instance, commonality analysis [64].

Until more systematic investigations are performed, we suggest that continuous phylogeography combined with univariate or bivariate approaches might be more appropriate and reliable for testing hypotheses concerning pathogen spread throughout natural landscapes, at least for the kind of sampling and geographical scales considered here.

### (f) Considerations for future work

Our study combined data of different BTV serotypes, topotypes (geographical variants within serotypes) and genome segments. While sample size limitations precluded us from conducting analyses at these finer levels, there are valid biological reasons why these could be important to consider. For example, some serotypes may not require a midge vector for transmission, which instead could happen via the placenta or through direct contact [65,66]. The importance of a given environmental driver for BTV diffusion might, thus, depend on the virus type or strain, as already discussed with respect to the effect of temperature (see above). Furthermore, the rate and mode of BTV diffusion across a landscape may differ among segments. Segments vary in their propensity for entering other strains through reassortment [17] and some segment variants might have an adaptive advantage in particular host or vector species. Finally, BTV transmission dynamics might differ between cattle/sheep breeds, or due to other forms of host heterogeneity, which could add geographical variation to spatial diffusion patterns. While ignoring these different aspects of variability, as we did here, is not expected to bias results, it will add statistical noise that could obscure biologically interesting relationships. Future work that examines potential sources of heterogeneity for their relevance to BTV spread would be valuable.

## 5. Conclusion

Our findings indicate that BTV spread occurs under the combined influence of climatic, landscape and host density factors, which will be useful for the development of better predictive models for BTV. Our results are also relevant to the spread of BTV vaccine strains, or their individual segments, given evidence that such strains have repeatedly undergone reassortment with field strains in Europe, followed by spatial dissemination [17]. More broadly, our work demonstrates how different phylogenetic and spatial approaches can be combined to gain insights into the ecological factors underlying pathogen diffusion and how this can be applied to the study of vector-borne diseases. In addition to these biological insights, the study highlights several important areas for methodological improvement that can currently limit robust inference of spatial transmission dynamics from pathogen genetic data. Addressing these limitations is timely, given the ongoing threat of disease emergence, creating an urgent need to better integrate molecular, spatial and epidemiological information to guide strategies for early warning, surveillance and control.

## Supplementary Material

Table S1

## Supplementary Material

Table S2

## Supplementary Material

Table S3

## Supplementary Material

Figure S1

## Supplementary Material

Figure S2

## Supplementary Material

Figure S3

## Supplementary Material

Figure S4

## Supplementary Material

Supplementary Data

## Data accessibility

Samples information and GenBank accession numbers of the 113 genomes used in this study are summarized in electronic supplementary material, table S1. Sources of environmental and meteorological data can be found in table 1 and rasters can be downloaded from https://figshare.com/s/61c7ae4b264d519933f8.
