# Supplementary figures and images for: Bluetongue virus spread in Europe is a consequence of climatic, landscape and vertebrate host factors as revealed by phylogeographic inference
Source: Proc Biol Sci. 2017 Oct 11;284(1864):20170919. doi: 10.1098/rspb.2017.0919 (PMC5647287; doi:10.1098/rspb.2017.0919)

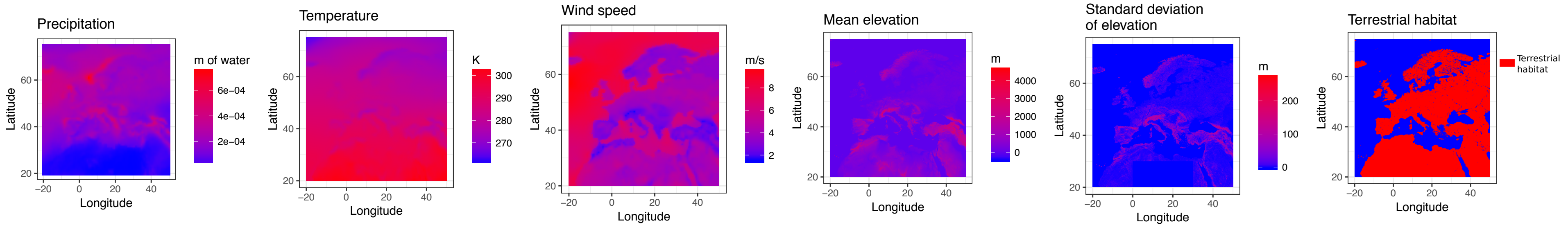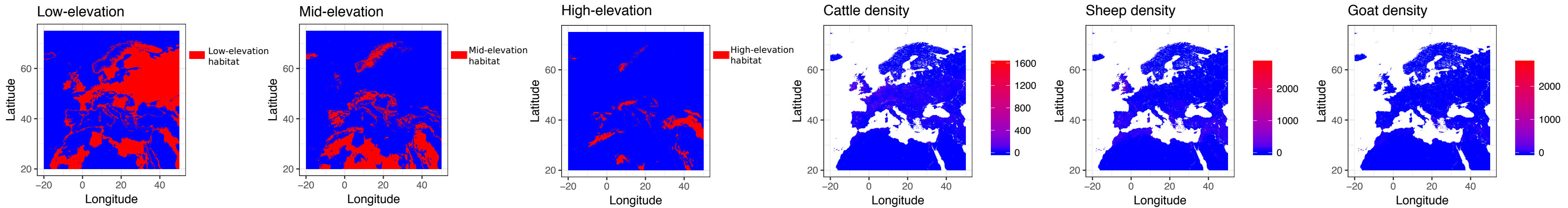

Supplement: Figure S1 [file rspb20170919supp4.pdf]

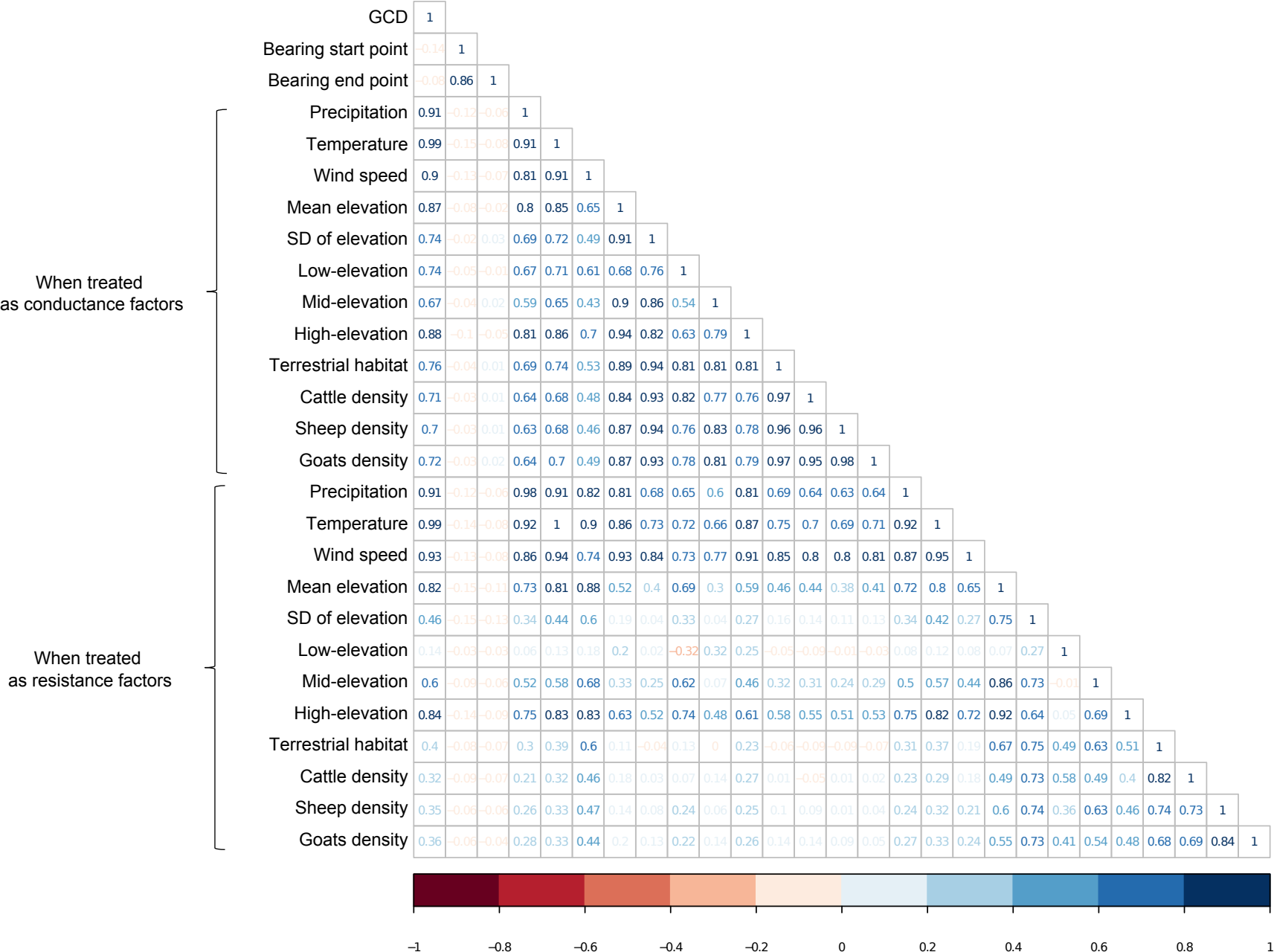

Supplement: Figure S2 [file rspb20170919supp5.pdf]

When treated  
as conductance factors

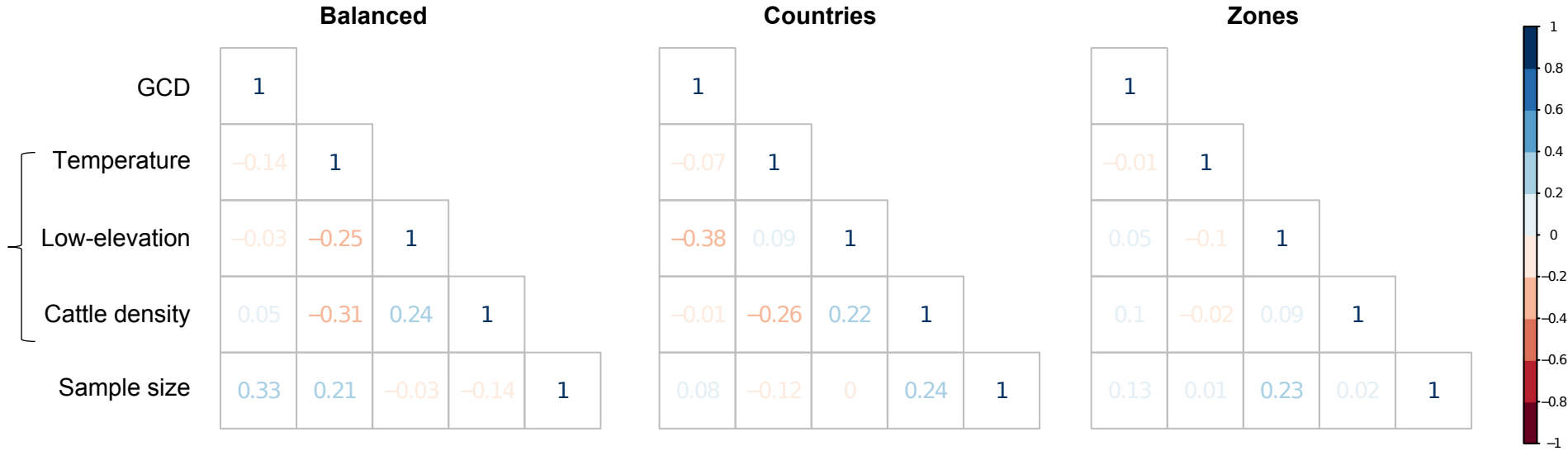

Supplement: Figure S3 [file rspb20170919supp6.pdf]

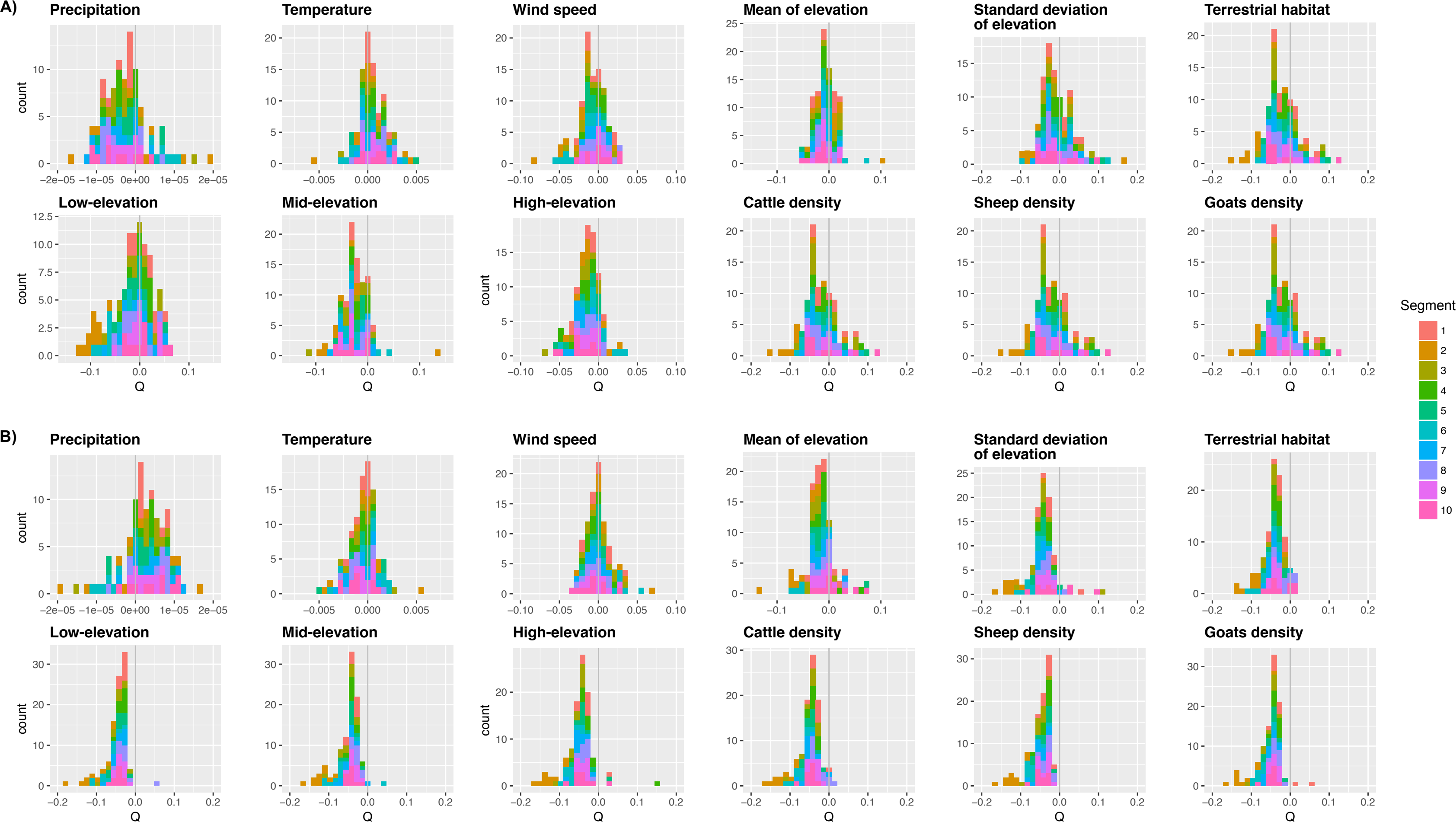

Supplement: Figure S4 [file rspb20170919supp7.pdf]
